# HIV Model Parameter Estimates from Interruption Trial Data including Drug Efficacy and Reservoir Dynamics

**DOI:** 10.1371/journal.pone.0040198

**Published:** 2012-07-16

**Authors:** Rutao Luo, Michael J. Piovoso, Javier Martinez-Picado, Ryan Zurakowski

**Affiliations:** 1 Department of Electrical and Computer Engineering, University of Delaware, Newark, Deleware, United States of America; 2 Department of Electrical Engineering, Pennsylvania State University Great Valley, Malvern, Pennsylvania, United States of America; 3 Institut de Recerca de la Sindrome de Inmunodeficencia Adquirida, IrsiCaixa, Badalona, Spain; 4 Instituci Catalana de Recerca i Estudis Avanats, Barcelona, Spain; National HIV and Retrovirology Laboratories, Canada

## Abstract

Mathematical models based on ordinary differential equations (ODE) have had significant impact on understanding HIV disease dynamics and optimizing patient treatment. A model that characterizes the essential disease dynamics can be used for prediction only if the model parameters are identifiable from clinical data. Most previous parameter identification studies for HIV have used sparsely sampled data from the decay phase following the introduction of therapy. In this paper, model parameters are identified from frequently sampled viral-load data taken from ten patients enrolled in the previously published AutoVac HAART interruption study, providing between 69 and 114 viral load measurements from 3–5 phases of viral decay and rebound for each patient. This dataset is considerably larger than those used in previously published parameter estimation studies. Furthermore, the measurements come from two separate experimental conditions, which allows for the direct estimation of drug efficacy and reservoir contribution rates, two parameters that cannot be identified from decay-phase data alone. A Markov-Chain Monte-Carlo method is used to estimate the model parameter values, with initial estimates obtained using nonlinear least-squares methods. The posterior distributions of the parameter estimates are reported and compared for all patients.

## Introduction

Many researchers have analyzed the dynamics of human immunodeficiency virus (HIV) using nonlinear ordinary differential equation (ODE) models [1]–[5]. Using various mathematical models, they sought to simulate the dynamics of the virus or to help design a treatment. Many of these studies have attempted to identify model parameters from patient data, but the sparsity of measurements resulted in very large confidence intervals in the parameter estimates. Furthermore, the experimental data used only included the period of viral decay following the introduction of therapy. Identification of the HIV model parameters under these conditions requires assuming that the drug efficacy is known, which in turn affects the estimates of the remaining parameters.

The amount of data normally available for HIV model identification is sparse. Typically, a patient on effective therapy has his or her viral load tested every 3 or 4 months, a rate too slow to accurately capture the dynamic characteristics accurately. In this paper, the data used to identify model parameters are from the AutoVac study conducted at the IrsiCaixa HIV research foundation in Barcelona [6]. In the AutoVac study, 12 patients underwent a series of about 30-day treatment interruptions, followed by resumption of suppressive therapy, and viral load measurements were taken at 3-day intervals during the interruption. This resulted in between 69 and 114 viral load measurements per patient, with between 38 and 77 data points per patient above the limit of detection. Therefore, data gathered from this particular experiment are sufficiently rich for model identification. All patients enrolled in the AutoVac study had maintained undetectable viral loads on therapy for at least two years prior to the study, and had baseline CD4+ T-Cell counts over 700. Patient nine and twelve both had interruptions without measurable viral load rebound, and were excluded from this study due to insufficient usable data.

Full-state measurements are not available for HIV model identification, so the problem of identifiability must be considered. For HIV models, Stafford et al. [7] have studied the identifiability of a 3 state model. Xia and Moog [8] analyzed the theoretical identifiability of a 4 state model and determined the minimal number of state measurements needed for estimating all model parameters. Frequently, only viral load data are available and in this case, not all parameters can be identified independently [7], [9]. Recently, Miao et al. also investigated some identifiability issues for viral dynamics [10].

**Figure 1:**
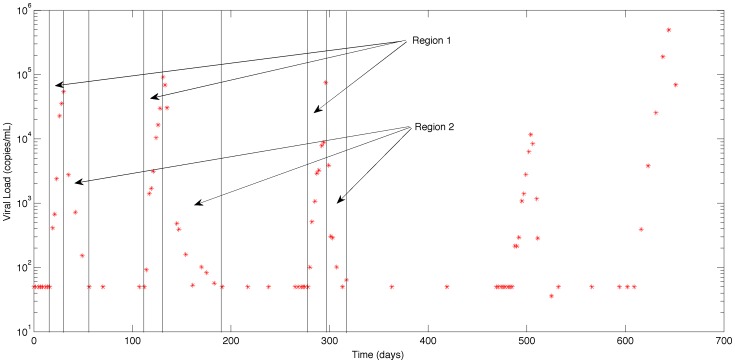
The viral load data for Patient 1.

For identifying the parameters of a viral dynamics model, two major methods are commonly used: nonlinear least squares [11] and Bayesian estimation [12]–[16]. In this paper, we employ a Bayesian Markov-Chain Monte Carlo technique as in [12], [16], with nonlinear least-squares used to generate initial conditions for the MCMC technique. The primary difference between this work and previous works is the quality of the data used for estimation. The data used in [16] consists of 10 measurements from 12 patients taken at 5 time points. The data used in [12] consists of seven measurements from 42 patients taken at seven time points. The data used in [15] consists of 9 viral load measurements from 42 patients taken at nine time points, plus a single baseline measurement of phenotypic drug susceptibility and survey data on patient adherence. The data used in [17] used 18 measurements of viral load from 18 time points. All four of these studies only included data from a single virus decay phase following treatment initiation. As a result of the sparse data, these previous studies had to make a number of simplifying assumptions about parameter values in order to preserve identifiability. By contrast, the AutoVac patient study provides us with between 69 and 114 viral load measurements from 10 patients from between 3 to 5 treatment interruptions cycles per patient. The high quality of the data used in this study allows reliable estimation of parameter values without resorting to the simplifying assumptions used in previous studies.

We consider the following mathematical model characterizing the viral dynamics for a patient:
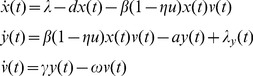
(1)


**Figure 2:**
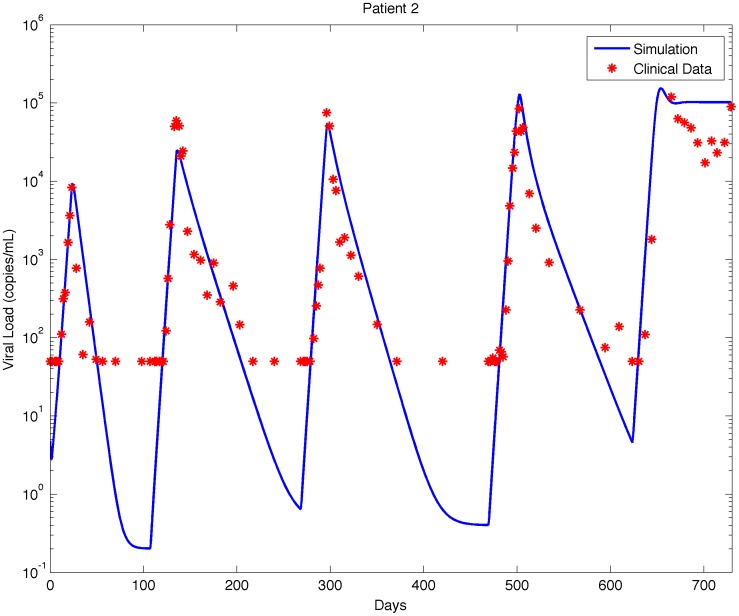
Model fitting for identified patients. Red star: experimental data (detection limit: 50 copies/mL); solid line: simulation based on estimated parameters.

There are three states: 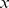, the concentration of target CD4+ T cells; 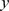, the concentration of actively infected CD4+ T cells; 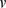, the viral load. 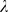 is the proliferation rate and 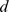 is the death rate of target CD4+ T cells; 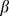 is the infection rate; 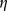 is the drug efficacy; 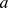 is the death rate of actively infected cells; 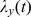 is the contribution of the reservoir to actively infected CD4+ T cells; 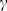 is the rate of free virus production by infected cells; 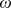 is the clearance rate for the free virus. The drug application 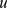 is 0 during interruptions and 1 during treatment. This is a variation of a model first proposed in [18], with the addition of the 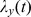 term describing the additional contribution of infected cells from all viral reservoir processes. This model is essentially the same as the model identified against patient data in the previous studies [11]–[15].

Highly active antiretroviral therapy (HAART) has proven effective to reduce the active viral load [19], [20] and is standard care for HIV patients. However, it cannot eradicate the virus completely [21], [22]. Although scientists suspect that the existence of long-term latent reservoirs in patients is the main reason for viral persistence [23], [24], there is little quantitative understanding of their contribution, mainly because of the difficulty measuring the virus reservoir directly. Some HIV investigators have proposed mathematical models to describe the dynamics of long-term latent reservoirs [25]–[27]. Little research has been done to estimate the parameters of these models quantitatively based on clinical data.

In this study, the total contribution of the reservoir processes to the actively infected CD4+T cells is estimated from standard viral-load time-series data. We analyze the identifiability for each parameter in Model 1 using differential algebra tools. The implementation of Bayesian estimation method is presented, and the joint posterior parameter distributions calculated by the Bayesian methods for each patient are reported.

**Figure 3:**
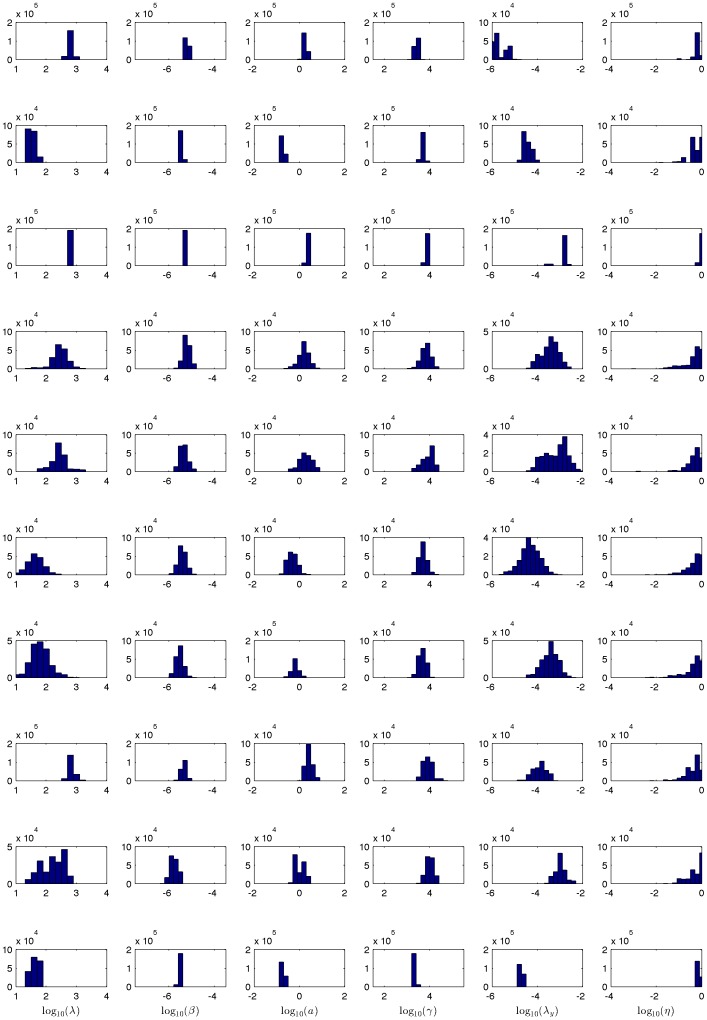
The marginal posterior distribution of each parameter for ten different patients.

## Methods

### Experimental Methods

#### Ethics statement

The previously published clinical study [6] was carried out in accordance with a human subjects protocol approved by the institutional ethics review committee at the University Hospital Germans Trias i Pujol in Barcelona, Spain. Written informed consent was obtained from all study participants. De-identified patient data was shared in accordance with a protocol approved by the University of Delaware Institutional Review Board.

**Table 1:** The average correlation coefficients between parameters for all ten patients.

	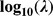	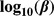	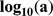	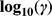
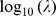	1	0.0953	0.6632	0.4278
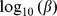	0.0953	1	0.1228	−0.6421
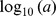	0.6632	0.1228	1	0.5817
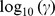	−0.6421	0.5817	0.6632	1

#### Study design

This research described here uses data from a previously published study. The measurements which are the focus of this work have been previously described in [6]. Briefly, a randomized prospective Structured Treatment Interruption study enrolled 26 HIV-1 positive asymptomatic adults with no detectable virus for at least two years prior to entering the study (limit of detection 50 virions per ml). 14 were randomized to a control group, continuing their previous cART regimens. 12 were randomized to the experimental group, and underwent between three and five cycles of interrupted antiviral therapy, remaining off therapy until two consecutive viral load measurements above 3000 virions/mL were reached, or for a maximum of 30 days, then re-initiating the original cART regimen for 90 days before the next interruption cycle began. HIV-1 RNA PCR quantitative analysis was performed on samples collected three times weekly following treatment interruption, and then weekly for the two months following re-initiation of treatment.

### Modeling Persistence of Latent Reservoirs

Although highly active antiretroviral therapy (HAART) efficiently suppresses viral load to undetectable levels, current regimens cannot eradicate the virus completely [21], [26], [28]–[30]. One possible reason is the persistent replication of HIV at a very low level, even under HAART conditions [26], [31], [32]. Another possible reason is the existence of stable reservoirs of latently infected cells [26], [33], [34]. These two possibilities are not mutually exclusive, and it is likely that a combination of persistent viremia and reservoir activation combine to maintain the reservoirs and prevent eradication [26].

Rong and Perelson proposed models to describe the dynamics of the latent reservoir [26]. However, in order to be identifiable from viral-load data, the models must be simplified. Siliciano et al. [35] found that the average half-life of the latent reservoir in resting CD4
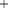
T cells is 44 months, which means it is extremely stable. There is no strong evidence that the activation rate of the reservoir is constant, however, and a combination of various factors may contribute to the maintenance of a residual virus load during effective antiviral suppression. Therefore, in Equation 1 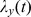 represents the total average contribution of reservoir dynamics to the active infected cell compartment 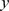 during the treatment period. The time between ceasing antiviral therapy and the viral load reaching measurable levels (the rebound time) is very sensitive to the value of 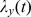, and consequently the goodness of fit for the entire model is also very sensitive to 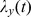. Surprisingly, as shown in our results, a patient-specific constant value for 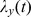 provided an excellent fit for all ten patients for all interruption cycles, implying that the average contribution of reservoir dynamics to the active infected cell compartment is relatively constant across several interruption cycles.

**Figure 4:**
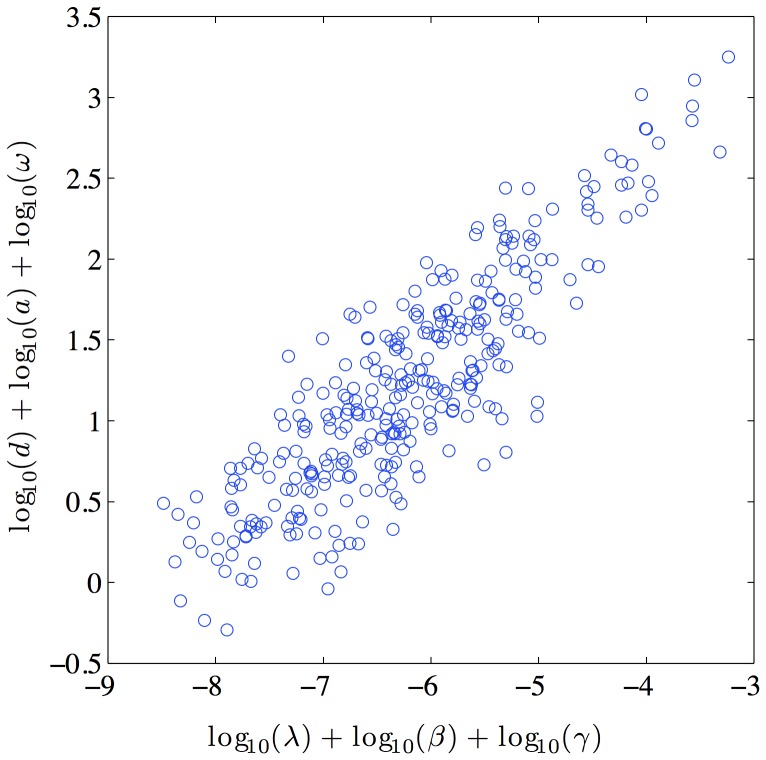
Typical scatter plot of *R*_0_ correlation. The heavy correlation between the elements in the numerator and denominator of *R*_0_ demonstrates the strong higher-order correlation between parameters.

**Table 2:** Parameter identification results for each patient, reported as mean(standard deviation).

Parameter	Unit	Patient 1	Patient 2
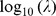	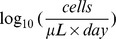	2.77(0.07)	1.54(0.12)
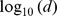	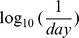	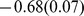	−1.33(0.12)
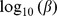	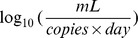	−5.26(0.09)	−5.48(0.06)
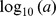	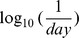	0.23(0.08)	−0.76(0.07)
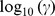	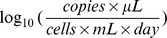	3.45(0.12)	3.69(0.05)
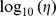	–	−0.14(0.18)	−0.10(0.28)
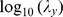	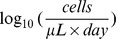	−5.67(0.30)	−4.43(0.17)
**Parameter**	**Unit**	**Patient 3**	**Patient 4**
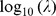	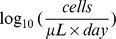	2.88(0.04)	2.45(0.27)
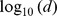	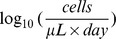	−0.34(0.04)	−0.61(0.27)
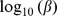	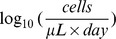	−5.35(0.01)	−5.23(0.15)
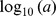	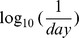	0.37(0.04)	0.19(0.24)
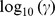	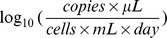	3.84(0.03)	3.82(0.22)
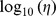	–	−0.05(0.03)	−0.17(0.43)
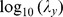	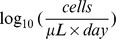	−2.94(0.20)	−3.45(0.38)
**Parameter**	**Unit**	**Patient 5**	**Patient 6**
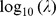	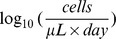	2.43(0.27)	1.64(0.29)
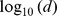	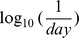	−0.64(0.27)	−1.28(0.29)
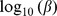	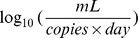	−5.36(0.16)	−5.42(0.18)
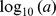	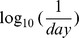	0.26(0.28)	−0.32(0.21)
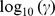	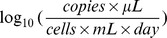	3.90(0.25)	3.70(0.17)
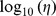	–	−0.17(0.37)	−0.15(0.38)
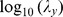	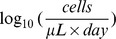	−3.18(0.51)	−4.33(0.43)
**Parameter**	**Unit**	**Patient 7**	**Patient 8**
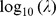	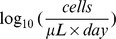	1.79(0.31)	2.83(0.11)
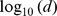	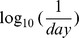	−1.33(0.31)	−0.44(0.11)
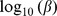	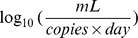	−5.54(0.18)	−5.36(0.12)
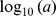	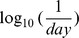	−0.18(0.15)	0.42(0.14)
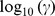	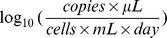	3.66(0.16)	3.91(0.21)
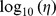	–	−0.17(0.42)	−0.22(0.36)
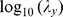	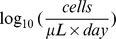	−3.46(0.36)	−3.90(0.31)
**Parameter**	**Unit**	**Patient 10**	**Patient 11**
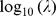	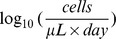	2.21(0.37)	1.65(0.10)
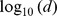	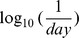	−0.96(0.37)	−1.35(0.10)
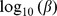	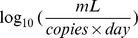	−5.78(0.17)	−5.54(0.04)
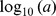	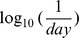	0.00(0.21)	−0.74(0.07)
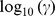	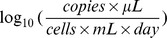	4.00(0.17)	3.39(0.01)
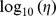	–	−0.11(0.34)	−0.13(0.06)
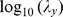	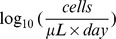	−3.00(0.24)	−4.65(0.09)

### Identifiability Analysis

Equation 1 is a special case of the following general nonlinear model:
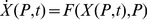
(2)


In this study 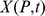 is the state vector 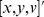, 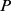 is the parameter vector 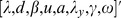, and 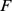 is the function which describes system dynamics.

We adapt the following concepts of identifiability from [36]–[38]:

**Definition 1.** Equation 2 is said to be globally identifiable from the given states if the equation 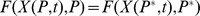 has only one solution 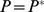.

**Definition 2.** Equation 2 is said to be locally identifiable from the given states if in some open neighborhood, 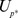, around the true parameter vector, the equation 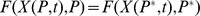 has only one solution 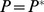 and 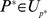.

**Table 3:** Result comparison between this paper and previous studies.

		Source		
		Luo et al.	Huang et al. [15]	Putter et al. [16]
		Average MLE	Posterior Mean	Posterior Median
		(Interpatient Range)	(95% CI)	(Interpatient Range)
Parameter	Units			
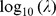	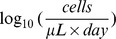	2.47	1.97	0.11
		(1.54, 2.88)	(1.93,2.05)	(−0.24,0.21)
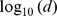	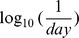	−0.74	−0.96	−2.99
		(−1.35, −0.34)	(−1.01, −0.40)	(NA)
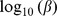	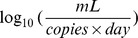	−5.41	−4	−6.80
		(−5.78, −5.23)	(−4.00, −3.15)	(−6.94, −6.53)
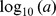	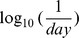	0.01	−0.42	−0.50
		(−0.76,0.42)	(−0.49, −0.22)	(−0.62,0.26)
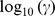	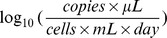	3.77	2.51	4.87
		(3.39,4.00)	(NA)	(4.83,4.89)
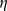	–	0.73	NA	0.71
		(0.60,0.89)	(NA)	(0.63,0.84)
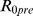	–	1.93	NA	3.90
		(1.16,3.68)	(NA)	(1.72,4.86)
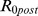	–	0.56	NA	0.34
		(0.13,0.76)	(NA)	(0.05,0.60)
# of patients		10	42	12
# of measurements per patient		98	9	5

The identifiability of HIV dynamic models has been analyzed previously [8], [10]; however, these previous works assumed more than one state could be measured. Here, only the viral load data are assumed to be available. Differential algebra is used to analyze the identifiability issue of Model 1. Details of the differential algebraic techniques are found in [37]–[39]. The steps in this analysis are as follows:

- We choose the following order relation for Equation 1: 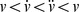.
- Based on the above order, the normalized characteristic polynomial of 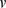 is calculated:
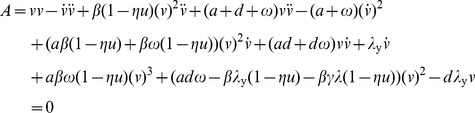
(3)

Equation 3 is generated by a) solving for 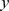 from the last equation of Equation 1, b) substituting for 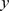 in the middle equation of Equation 1, c) solving for 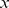 from that equation and d) substituting for 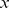 in the first equation of Equation 1. Knowledge of 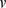 allows one to estimate the coefficients in Equation 3. This characteristic polynomial does not contain the states 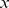, 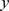 and their derivatives, but still describes the viral dynamics of Equation 1.

- iii) By extracting the coefficients in the above polynomial we obtain the following set of identifiable parameters:

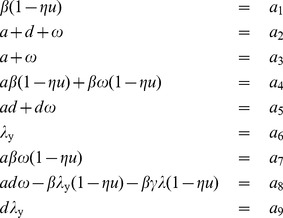
(4)iv) The identifiability of each parameter can be checked by checking the injectivity of the the map defined in Equation 4. All parameters except 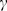 and 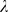 are uniquely identifiable; the product 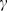 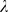 is uniquely identifiable. Since the patients enrolled in the AutoVac study had all maintained undetectable viral loads on therapy for at least two years prior to the study, it is reasonable to assume that the initial conditions of this study are the steady states in Equation 1. Therefore, the steady state of 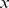 in Equation 1, 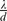, is set as the concentration of CD4+ T Cells at the beginning of this study. Under this assumption, the value of 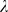 is defined as the product of the initial number of target cells 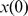 and the estimated decay rate of target cell 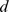.

Although theoretically 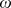 is uniquely identifiable, the current best estimate of 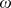 is between 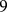 and 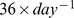 [40]. Estimation of 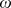 would require very high frequency measurements (several measurements per hour, much faster than our data). Therefore the value of 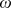 is set as 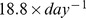 (corresponding to the geometric mean of the estimated half-lives in [40]), and the virus dynamics are treated as a singular perturbation to the system. Assuming that the true value of 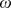 is sufficiently large that the singular perturbation approximation is valid, the only identified parameter that depends on this assumption is 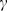; the posterior distribution of estimated values for 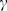 would shift in a inversely proportional manner with a change in the assumed value of 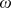, with no change in the posterior distributions of any other parameter.

The identifiability analysis described above does not take into account the number of measurements or the sampling rate; it simply states that for a sufficiently large number of measurements taken at a sufficiently fast sampling rate, the set of parameters described are identifiable. The uncertainty in the estimated parameter values will depend on the actual number of measurements and their frequency.

### Bayesian Estimation

#### Initial estimates

During the AutoVac study treatment for each patient was interrupted and after a period of time, restarted. This cycle of interruption and reinstating the treatment is repeated 3 to 5 times. In order to generate an acceptable initial value for the MCMC method, a two-step least-squares method was used, using data from the first three interruption cycles. The data from the period in which the treatment is interrupted, region 1, is used to estimate the 5 parameters, 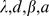 and 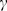 using a constrained nonlinear least squares method. The data from region 2, where treatment is reinstated, contains information about the drug efficacy. With the value of the six parameters fixed to those values found by least squares in region 1 (as shown in Fig. 1), the data of region 2 is used to estimate the drug efficacy. The initial values obtained using this two-step procedure are used as the kernel for the MCMC methodology described below; the MCMC methodology identifies all six parameters against all data from both regions of all treatment interruptions.

**Figure 5:**
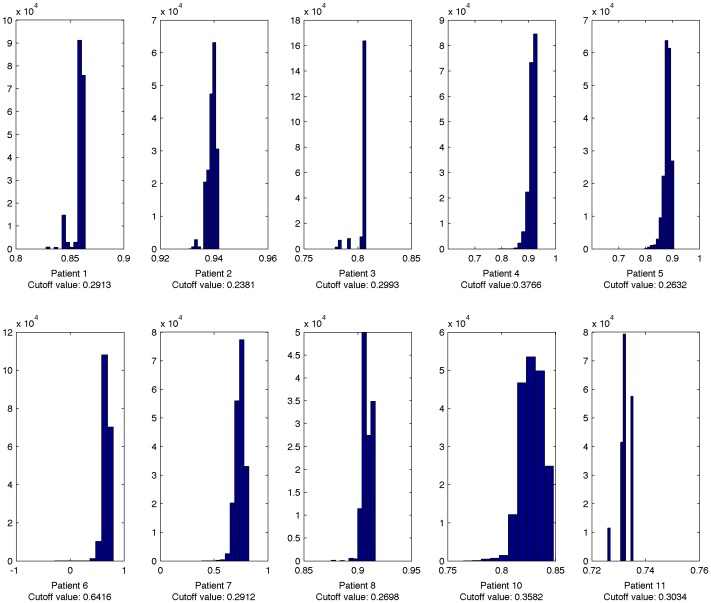
The histogram of coefficient of determination. *R* ^2^ Cutoff values: The value of *R* ^2^ corresponding to *P* = 0.05.

**Figure 6:**
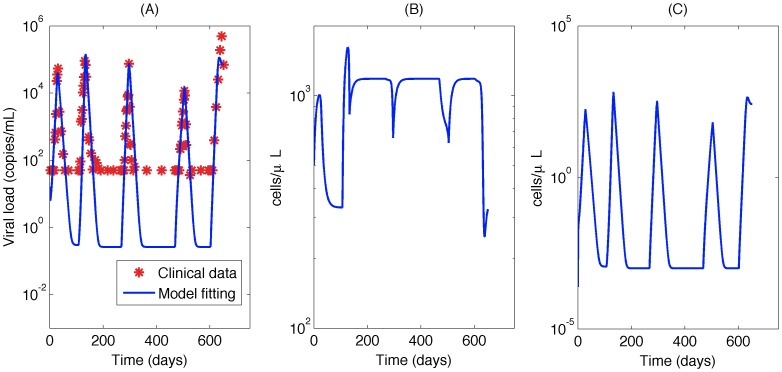
Typical Bayesian Posterior Mean Results. (A)The fitted viral load curve and viral load data for patient 1; (B)Target cell simulation using fitted parameters; (C)Infected cell simulation using fitted parameters.

#### MCMC methodology

From the steady-state values of Equation 1, the relationship between 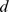 and 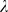 can be written as:
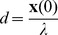
(5)


Where 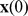 is the initial measurement of CD4+ T cells taken for each patient at the beginning of the study (as in [16]). Therefore, in this method, the parameter set 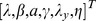 are estimated. 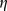 is calculated for each iteration by least-squares subject to the values of the other five parameters. The MCMC approach taken here is based on the Metropolis-Hasting algorithm [13]. Assume that the 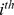 subject, we have 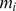 measurements of viral load. We denote the parameters as:
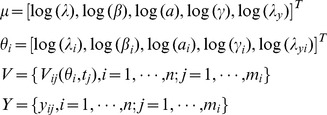



The logarithm is used to ensure that all estimates of the parameters are positive. The vectors, 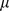 and 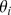 are the logarithm of the parameters for the population level and the logarithm of the parameters for the 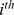 individual respectively. The initial values of 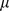 and 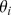 are set as the results of Patient 2 from the nonlinear least-square method described above, which are 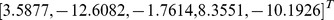. The matrix 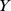 is the matrix of the logarithm of available measurements to base 10 for all the patients. Let 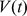 denote the solution of the differential equation and 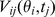 denote the value of 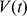 for the 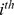 patient using parameters 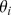 at time 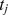.

Following the iterative MCMC algorithm of [13], the implementation can be written as:

1. Initialize the chain with initial values 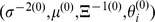.
2. Use Gibbs sampling steps to update 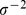, 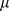, and 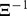 and use Metropolis-Hastings algorithm to update 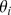:
  (a) 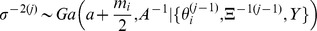;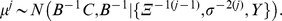.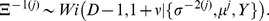.

where 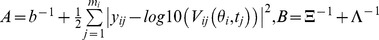, 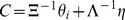 and 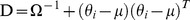.


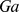
 is the gamma distribution and 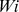 is the Wishart prior distribution. The hyper-parameters, 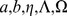 and 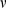 are known with the following values:
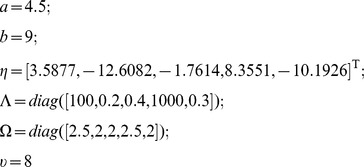



The hyper-prior values for the initial variance of 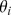, 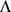, are sufficiently large that this may be considered a non-informative prior distribution. For comparison, the analysis in [16] had initial variance for 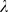 and 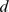 of 0.12 and 0.016 respectively, heavily biasing the posteriors to the priors, and the analysis in [15] had initial variance for the parameters 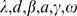 of 0.005, also substantially biasing the posterior distributions of these parameter estimates to the prior distributions.

(b) Generate a prospective value 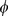 for 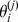 from the previous iterative value 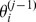 where

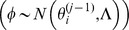

(c) Evaluate the acceptance probability of this move by applying Metropolis-Hastings algorithm. If this move is accepted, 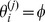. If not, 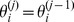.
3. Repeat step 2 until the chain converges.

To obtain reasonable results from the MCMC method, good initial estimates of 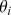 are needed. The constrained least squares approach described previously is used to get an initial estimate. The parameters are constrained so that 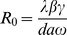, which is the basic reproduction ratio during treatment interruptions, is greater than 1. If 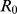 were less than 1, then the virus would not have successfully established infection.

The above procedure was applied to the data for 10 patients with sufficient data. The MCMC procedure produced 200,000 possible sets of parameters for each patient that are consistent with the patients’ data. For the purposes of analysis, the first 50,000 iterations were discarded to allow the chain to converge, leaving 150,000 parameter sets per patient for the final analysis. From this result, the marginal probability densities for of the six parameters can be established.

## Results

### Nonlinear Least Squares Estimation

Parameter estimates were generated for each of 10 patients using the nonlinear least squares method. Of the 12 patients in the study, 2 had no detectable virus after an interruption, leaving insufficient data above the measurement threshold to identify model parameters. Although the nonlinear least-square method cannot guarantee globally optimal results, it can provide good initial estimates for the prior distribution of the MCMC method. Simulated viral load curves based on the identified parameter values using this method are compared to the measured data from Patient 2 in Fig. 2.

### Bayesian Estimation

The MCMC model fitting procedure was run for each of the 10 patients with sufficient data. Histograms of the marginal posterior distributions for the six parameters for each of the 10 patients are shown in Fig. 3.

Note that the parameters are not independent, and the parameter vectors should be considered as complete sets. Table 1 shows the average correlation coefficient among each different pair of parameters; it is clear that most pairs of parameters would be considered highly correlated. In addition to this first-order correlation between parameters, there are also higher order nonlinear correlations. Fig. 4 shows the high level of correlation between the product of the parameters which form the numerator and denominator of 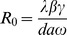, further emphasizing the need to consider parameter vectors rather than individual parameters. Values chosen independently from each parameter’s distribution can easily generate a parameter set which is a particularly poor representation of the data. Consequently, we also report as tables in the supplementary material the entire posterior distribution for each of the 10 patients (shown in supplementary tables S1, S2, S3, S4, S5, S6, S7, S8, S9, S10). The posterior distribution generated by this MCMC method provides a database for testing the robustness of treatment optimization strategies, such as those described in [25], [41]–[43].

The histograms in Fig. 3 show the range of values of each parameter, and Table 2 gives the maximum likelihood estimate for each parameter. From the histograms it is clear that the distributions for the parameters 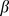,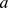, and 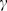 vary little between patients, indicating that the infection rate and burst size of the virus and the death rate of infected cells may be consistent across the patient population. By contrast, the parameters 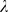, 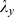, and 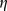 vary substantially between patients, indicating that the regeneration rate of CD4+ T cells, the reservoir contribution rate, and the drug efficacy may vary significantly across the patient population.

Table 3 gives a summary of the estimated population parameters (the average value of the 10 identified patients), compared with those from previously published papers [15], [16]. 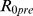 is the basic reproductive ratio 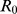 of the virus without treatment. 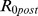 is 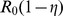, the reproductive ratio of the virus under treatment conditions. The values for parameters 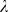,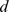,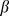,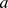, and 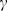 are consistent with the previously published best estimates for these parameters. In particular, the maximum likelihood estimates for the death rate of target cells 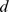 ranged from 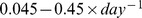, in perfect agreement with the estimates obtained from patient data in [15]. This estimate is considerably higher than the decay rate estimated in [16], but careful examination of the methods in that previous work show that the value of 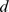 was essentially constrained to the prior distribution. The ratio 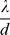 is consistent between all three studies. Our maximum likelihood estimates for the death rate of infected cells 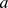 ranged from 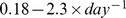, in agreement with the current best estimate of 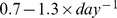 [17], [26], as well as the data from the two comparable studies [15], [16]. Our maximum likelihood estimate of the density-dependent infection rate 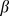 ranged from 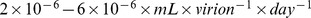, compared to 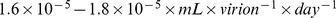 [13] and 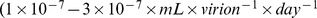 [16]. Our maximum likelihood estimates of the target cell recruitment rate 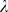 ranged from 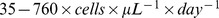, higher than the comparable range of 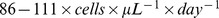 reported in [15]; however, this is expected, as the inclusion criteria for our experiment resulted in patients with much healthier immune systems overall compared to the patients in [15]. The much lower rate of 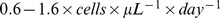 reported in [16] is a direct result of that study’s unrealistically low prior value for 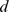. Our estimates of the virus production rate 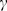 range from 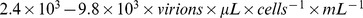, higher than the 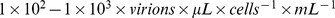 range reported in [15]; however, this is due to the estimate in [15] of 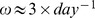, as opposed to our fixed estimate of 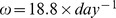.

The maximum likelihood values of the reservoir contribution rate ranged over three orders of magnitude, from 
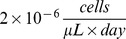
 for Patient 1 to 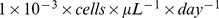 for Patients 3 and 10. This suggests that the replenishment of the active compartment by the viral reservoirs is very heterogenous between patients. However, it is noteworthy that a single constant value of 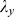 was able to accurately predict rebound time across multiple interruption cycles for the same patient despite variation in interruption length, indicating that the replenishment rate for a given patient is relatively constant over the course of the experiment.

The efficacy of the antiviral drugs is estimated directly from the viral load data. To our knowledge, this is the first time this has been done. Previous estimates of model parameters have typically inferred drug efficacy from PK/PD data in the plasma, resulting in estimates of 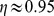 [15], or relied heavily on the assumption that the first measurement was at steady-state [16], resulting in estimates between 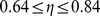. By contrast, our direct estimate of 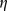 from the viral load data give us maximum likelihood estimates ranging from 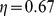 to 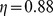. This indicates that the estimates based on plasma pharmacokinetic data may overestimate the true drug efficacy, though the estimates which relied exclusively on the first measurement were consistent with our results.

Fig. 5 shows the histograms of the coefficient of determination 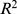 for each of the 10 patients. The statistical significance threshold for the fit relative to an average measurement model was calculated using the F-test (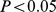 is considered statistically significant); this shows that, for all patients except Patient 6, all 150,000 parameter vectors in the posterior distribution would be considered a statistically significant fit to the data if considered in isolation.

The viral load fitted by the model using the maximum likelihood estimates of the parameters and simulation results for Patient 1 are shown in Fig. 6.

## Discussion

As shown in this study, the parameters in the HIV dynamics model are heavily correlated. The strong correlation between parameters in this model mean that only the distribution of complete parameter sets (as opposed to independent distributions of each parameter) can be considered to accurately represent the fit of the model to the data. The quality and extent of the data available in this study was considerably higher than any previously published parameter estimation study, allowing for the accurate estimation of the model parameters without using the simplifying assumptions necessitated by the sparsity of data in previous studies. Furthermore, the level of excitation of the dynamics provided by the multiple interruption schedules allowed us to directly identify two parameters not previously identifiable from patient data. The results agree for the most part with previously published data, but the results are more reliable and significant given the dramatic increase in the amount of data available for identification.

This paper presents, for the first time, the posterior distribution of parameters for a commonly used HIV infection model identified against measured patient data. Analysis of this distribution shows good representation of the data. As shown in the histograms of Fig. 5, all parameter estimates in the posterior distributions for all patients except a subset of patient 6 would be considered statistically significant by the standard F Test (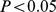), implying that the reported posterior distribution describes the range of feasible parameter values based on the measured data well. Inspection of the data from patient 6 shows the data available above the limit of detection are limited compared to the other patients, which can explain the high value of 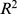 corresponding to an F-test P value of 0.05 for that patient. However, even for this patient, more than half of estimates are still statistically significant, and there were 39 measurement points above the limit of detection, substantially more measurements than were available for any previously published parameter estimation study.

The multiple interruptions in the patient data provided the opportunity to quantify the contribution rate of viral reservoirs to the active infected cell compartment. These rates varied widely from patient to patient. The rate was characterized in terms of number of productively infected cells produced per day, and equally well describes such diverse potential reservoir processes as low-level persistent replication, viral blipping, and spontaneous reactivation of quiescent cells. The overall fit is highly sensitive to the rebound time, and the rebound time is uniquely determined by the reservoir contribution rate. It is surprising, therefore, that a single value for the reservoir contribution rate was able to describe the data well over multiple interruptions in the same patient; this strongly suggests that the underlying process represented by the parameter 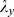 is continuous rather than bursting in nature. The quantification and understanding of the viral reservoir dynamics is of critical importance to understanding the nature of ongoing viral evolution under conditions of effective suppression, and will be a necessary precursor to any attempts to flush the reservoirs and achieve a functional cure for HIV.

The results presented here also show that the overall drug efficacy is between 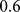 and 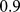, and that the effective reproductive ratio of the virus while on therapy is between 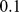 and 0.8, verifying the results presented in [16]. It is important to emphasize that, while the antiviral drug regimens are very successful at suppressing overall virus load, they are not perfect in inhibiting infection, and *de novo* infection events continue to occur even under highly effective therapy. The results presented here also verify the previously reported findings in [15] that uninfected CD4+ target cells have a half-life of approximately 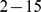 days *in vivo*, which is substantially shorter than estimates obtained *in vitro*. This is likely due to the higher state of activation of the CD4+ T-cells during active viremia *in vivo*, but whatever the cause, it is a consistent finding across multiple experiments.

In addition to being the most accurate way of describing the fit of a model to data with high levels of measurement uncertainty, the publication of complete parameter distributions identified from patient data also has significant practical importance. A growing number of model-based interventions using variations of the model described in Equation 1 are being proposed, including our own methods designed to minimize the risk of resistance emerging during antiviral regimen switching [25], [41], [42], [44]–[47]. Most of these methods have used either nominal parameters or a single parameter set. The parameter distributions published in this work provide a parameter set against which the robustness of a proposed model-based method to expected patient variation can be tested. The data used in this paper came from a cohort restricted to patients with good immunological control of the virus under antiviral suppression, so the distribution of parameters can only be said to be representative of such a subgroup of HIV-infected persons. However, the publication of the identification methods will likely lead to the publication of parameter distributions from patients in other studies in the future, leading to a growing library of virtual patients.

## Supporting Information

Table S1 **The posterior distribution {** ***θ_i_*** **} for Patient 1.** The first 50,000 rows were excluded from analysis to allow for convergence of the Markov Chain.
(XLSB)

Table S2 **The posterior distribution {** ***θ_i_*** **} for Patient 2.** The first 50,000 rows were excluded from analysis to allow for convergence of the Markov Chain.
(XLSB)

Table S3 **The posterior distribution {** ***θ_i_*** **} for Patient 3.** The first 50,000 rows were excluded from analysis to allow for convergence of the Markov Chain.
(XLSB)

Table S4 **The posterior distribution {** ***θ_i_*** **} for Patient 4.** The first 50,000 rows were excluded from analysis to allow for convergence of the Markov Chain.
(XLSB)

Table S5 **The posterior distribution {** ***θ_i_*** **} for Patient 5.** The first 50,000 rows were excluded from analysis to allow for convergence of the Markov Chain.
(XLSB)

Table S6 **The posterior distribution {** ***θ_i_*** **} for Patient 6.** The first 50,000 rows were excluded from analysis to allow for convergence of the Markov Chain.
(XLSB)

Table S7 **The posterior distribution {** ***θ_i_*** **} for Patient 7.** The first 50,000 rows were excluded from analysis to allow for convergence of the Markov Chain.
(XLSB)

Table S8 **The posterior distribution {** ***θ_i_*** **} for Patient 8.** The first 50,000 rows were excluded from analysis to allow for convergence of the Markov Chain.
(XLSB)

Table S9 **The posterior distribution {** ***θ_i_*** **} for Patient 10.** The first 50,000 rows were excluded from analysis to allow for convergence of the Markov Chain.
(XLSB)

Table S10 **The posterior distribution {** ***θ_i_*** **} for Patient 11.** The first 50,000 rows were excluded from analysis to allow for convergence of the Markov Chain.
(XLSB)

## References

[1] Wei X, Ghosh SK, Taylor ME, Johnson VA, Emini EA (1995). Viral dynamics in human immunodeficiency virus type 1 infection.. Nature.

[2] Ho DD, Neumann AU, Perelson AS, Chen W, Leonard JM (1995). Rapid turnover of plasma virions and CD4 lymphocytes in HIV-1 infection.. Nature.

[3] Nelson GW, Perelson AS (1995). Modeling defective interfering virus therapy for AIDS: conditions for HIV survival.. Math Biosci.

[4] Perelson AS, Neumann AU, Markowitz M, Leonard JM, Ho DD (1996). HIV-1 dynamics in vivo: virion clearance rate, infected cell life-span, and viral generation time.. Science.

[5] Bortz DM, Nelson PW (2006). Model selection and mixed-effects modeling of HIV infection dynamics.. Bull Math Biol.

[6] Ruiz L, Carcelain G, Martnez-Picado J, Frost S, Marfil S (2001). HIV dynamics and t-cell immunity after three structured treatment interruptions in chronic HIV-1 infection.. AIDS.

[7] Stafford MA, Corey L, Cao Y, Daar ES, Ho DD (2000). Modeling plasma virus concentration during primary HIV infection.. J Theor Biol.

[8] Xia X, Moog CH (2003). Identifiability of nonlinear systems with application to HIV/AIDS models.. IEEE T Automat Contr.

[9] Wu H, Zhu H, Miao H, Perelson AS (2008). Parameter identifiability and estimation of HIV/AIDS dynamic models.. Bull Math Biol.

[10] Miao H, Dykes C, Demeter LM, Wu H (2009). Differential equation modeling of HIV viral fitness experiments: model identification, model selection, and multimodel inference.. Biometrics.

[11] Liang H, Miao H, Wu H (2010). Estimation of constant and time-varying dynamic parameters of HIV infection in a nonlinear differential equation model.. Ann Appl Stat.

[12] Wu H, Huang Y, Acosta EP, Rosenkranz SL, Kuritzkes DR (2005). Modeling long-term HIV dynamics and antiretroviral response: effects of drug potency, pharmacokinetics, adherence, and drug resistance.. J Acquir Immune Defic Syndr.

[13] Huang Y, Liu D, Wu H (2006). Hierarchical bayesian methods for estimation of parameters in a longitudinal HIV dynamic system.. Biometrics.

[14] Huang Y (2008). Long-term HIV dynamic models incorporating drug adherence and resistance to treatment for prediction of virological responses.. Computational Statistics & Data Analysis.

[15] Huang Y, Wu H, Acosta EP (2010). Hierarchical bayesian inference for HIV dynamic differential equation models incorporating multiple treatment factors.. Biom J.

[16] Putter H, Heisterkamp SH, Lange JMA, de Wolf F (2002). A Bayesian approach to parameter estimation in HIV dynamical models.. Stat Med.

[17] Markowitz M, Louie M, Hurley A, Sun E, Di Mascio M (2003). A novel antiviral intervention results in more accurate assessment of human immunodeficiency virus type 1 replication dynamics and T-cell decay in vivo.. J Virol.

[18] Perelson A (1993). Dynamics of HIV infection of CD4+ T cells.. Mathematical Biosciences.

[19] Perelson AS, Essunger P, Cao Y, Vesanen M, Hurley A (1997). Decay characteristics of HIV-1-infected compartments during combination therapy.. Nature.

[20] Gulick RM, Mellors JW, Havlir D, Eron JJ, Gonzalez C (1997). Treatment with indinavir, zidovudine, and lamivudine in adults with human immunodeficiency virus infection and prior antiretroviral therapy.. N Engl J Med.

[21] Dornadula G, Zhang H, VanUitert B, Stern J, Livornese L (1999). Residual HIV-1 RNA in blood plasma of patients taking suppressive highly active antiretroviral therapy.. JAMA.

[22] Palmer S, Wiegand AP, Maldarelli F, Bazmi H, Mican JM (2003). New real-time reverse transcriptase-initiated PCR assay with single-copy sensitivity for human immunodeficiency virus type 1 RNA in plasma.. J Clin Microbiol.

[23] Finzi D, Hermankova M, Pierson T, Carruth LM, Buck C (1997). Identification of a reservoir for HIV-1 in patients on highly active antiretroviral therapy.. Science.

[24] Chun TW, Stuyver L, Mizell SB, Ehler LA, Mican JA (1997). Presence of an inducible HIV-1 latent reservoir during highly active antiretroviral therapy.. Proc Natl Acad Sci U S A.

[25] Luo R, Piovoso M, Zurakowski R (2009). A generalized multi-strain model of HIV evolution with implications for drug-resistance management.. In: Proc. American Control Conference..

[26] Rong L, Perelson AS (2009). Modeling latently infected cell activation: viral and latent reservoir persistence, and viral blips in HIV-infected patients on potent therapy.. PLoS Comput Biol.

[27] Rong L, Perelson AS (2009). Modeling HIV persistence, the latent reservoir, and viral blips.. Journal of Theoretical Biology.

[28] Smith DM, Wong JK, Shao H, Hightower GK, Mai SHT (2007). Long-term persistence of transmitted HIV drug resistance in male genital tract secretions: implications for secondary transmission.. J Infect Dis.

[29] Gunthard HF, Havlir DV, Fiscus S, Zhang ZQ, Eron J (2001). Residual human immunodeficiency virus (HIV) type 1 RNA and DNA in lymph nodes and HIV RNA in genital secretions and in cerebrospinal uid after suppression of viremia for 2 years.. J Infect Dis.

[30] Palmer S, Maldarelli F, Wiegand A, Bernstein B, Hanna GJ (2008). Low-level viremia persists for at least 7 years in patients on suppressive antiretroviral therapy.. Proc Natl Acad Sci U S A.

[31] Blankson JN, Persaud D, Siliciano RF (2002). The challenge of viral reservoirs in HIV-1 infection.. Annu Rev Med.

[32] Schrager LK, D’Souza MP (1998). Cellular and anatomical reservoirs of HIV-1 in patients receiving potent antiretroviral combination therapy.. JAMA.

[33] Chun TW, Carruth L, Finzi D, Shen X, DiGiuseppe JA (1997). Quantification of latent tissue reservoirs and total body viral load in HIV-1 infection.. Nature.

[34] Chun TW, Finzi D, Margolick J, Chadwick K, Schwartz D (1995). In vivo fate of HIV-1-infected T cells: quantitative analysis of the transition to stable latency.. Nat Med.

[35] Siliciano JD, Kajdas J, Finzi D, Quinn TC, Chadwick K (2003). Long-term follow-up studies confirm the stability of the latent reservoir for HIV-1 in resting CD4+ T cells.. Nat Med.

[36] Ljung L, Glad S (1994). On global identifiability for arbitrary model parameterizations.. Automatica.

[37] Saccomani MP, Audoly S, D’Angio L (2003). Parameter identifiability of nonlinear systems: the role of initial conditions.. Automatica.

[38] Saccomani MP, Audoly S, Bellu G, D’Angio L (2010). Examples of testing global identifiability of biological and biomedical models with the daisy software.. Computers in Biology and Medicine.

[39] Ritt JF (1950). Differential algebra.. Providence, R.I.: American Mathematical Society.

[40] Ramratnam B, Bonhoeffer S, Binley J, Hurley A, Zhang L (1999). Rapid production and clearance of HIV-1 and hepatitis C virus assessed by large volume plasma apheresis.. The Lancet.

[41] Luo R, Zurakowski R (2008). A new strategy to decrease risk of resistance emerging during therapy switching in HIV treatment.. In: Proc. American Control Conference..

[42] Luo R, Cannon L, Hernandez J, Piovoso M, Zurakowski R (2011). Controlling the evolution of resistance.. J Process Control.

[43] Luo R, Piovoso MJ, Martinez-Picado J, Zurakowski R (2011). Optimal Antiviral Switching to Minimize Resistance Risk in HIV Therapy.. PLoS ONE.

[44] Martinez-Cajas JL, Wainberg MA (2008). Antiretroviral therapy : optimal sequencing of therapy to avoid resistance.. Drugs.

[45] Cardozo EF, Zurakowski R (2012). Robust Closed-Loop Minimal Sampling Method for HIV Therapy Switching Strategies.. Biomedical Engineering, IEEE Transactions on 59: In press.

[46] Ferreira J, Hernandez-Vargas EA, Middleton RH (2011). Computer simulation of structured treatment interruption for HIV infection.. Comput Methods Programs Biomed.

[47] Hernandez-Vargas E, Colaneri P, Middleton R, Blanchini F (2011). Discrete-time control for switched positive systems with application to mitigating viral escape.. International Journal of Robust and Nonlinear Control.

